# Psychosocial working conditions, trajectories of disability, and the mediating role of cognitive decline and chronic diseases: A population-based cohort study

**DOI:** 10.1371/journal.pmed.1002899

**Published:** 2019-09-16

**Authors:** Kuan-Yu Pan, Weili Xu, Francesca Mangialasche, Rui Wang, Serhiy Dekhtyar, Amaia Calderón-Larrañaga, Laura Fratiglioni, Hui-Xin Wang

**Affiliations:** 1 Aging Research Center, Department of Neurobiology, Care Sciences and Society, Karolinska Institutet and Stockholm University, Stockholm, Sweden; 2 Division of Clinical Geriatrics, Center for Alzheimer Research, Department of Neurobiology, Care Sciences and Society, Karolinska Institutet, Stockholm, Sweden; 3 Stockholm Gerontology Research Center, Stockholm, Sweden; 4 Stress Research Institute, Stockholm University, Stockholm, Sweden; MRC Lifecourse Epidemiology Unit, UNITED KINGDOM

## Abstract

**Background:**

Unfavorable psychosocial working conditions have been associated with cognitive decline and chronic diseases, both of which may subsequently accelerate functional dependence. This study aimed to investigate the association between job demand–control–support combinations and trajectories of disability in later life and to further explore the role of cognitive decline and the co-occurrence of chronic diseases in mediating this association.

**Methods and findings:**

In this cohort study, 2,937 community dwellers aged 60+ years (mean age 73 ± 10.6; 62.9% female) residing in the Kungsholmen District of Stockholm, Sweden, participated in the baseline survey (2001–2004) and were followed up to 12 years. Lifelong occupational history was obtained through a standardized interview; job demands, job control, and social support at work in the longest-held occupation were graded with a psychosocial job–exposure matrix. Job control, demands, and social support were dichotomized using the median values from the matrix, respectively, to further generate demand–control–support combinations. Disability was measured by summing the number of impaired basic and instrumental activities of daily living. Global cognitive function was assessed by Mini-Mental State Examination. Chronic conditions were ascertained by clinical examinations, medical history, and patient clinical records; the total number of chronic diseases was summed. Data were analyzed using linear mixed-effects models and mediation analysis. Age, sex, education, alcohol consumption, smoking, leisure activity engagement, early-life socioeconomic status, occupational characteristic and physical demands, and baseline cognitive function and number of chronic diseases were adjusted for in the analyses. Compared with active jobs (high control/high demands; *n* = 1,807), high strain (low control/high demands; *n* = 328), low strain (high control/low demands; *n* = 495), and passive jobs (low control/low demands; *n* = 307) were all associated with a faster rate of disability progression (β = 0.07, 95% CI 0.02–0.13, *p* = 0.01; β = 0.10, 95% CI 0.06–0.15, *p <* 0.001; β = 0.11, 95% CI 0.05–0.18, *p <* 0.001). The association between high strain and disability progression was only shown in people with low social support at work (β = 0.13, 95% CI 0.07–0.19, *p <* 0.001), but not in those with high social support (β = 0.004, 95% CI −0.09 to 0.10, *p* = 0.93). Moreover, we estimated that the association between demand–control status and disability trajectories was mediated 38.5% by cognitive decline and 18.4% by accumulation of chronic diseases during the follow-up period. The limitations of this study include unmeasured confounding, self-reported work experience, and the reliance on a psychosocial job–exposure matrix that does not consider variabilities in individuals’ perception on working conditions or job characteristics within occupations.

**Conclusions:**

Our findings suggest that negative psychosocial working conditions during working life may accelerate disability progression in later life. Notably, social support at work may buffer the detrimental effect of high strain on disability progression. Cognitive decline and chronic-disease accumulation, and especially the former, partially mediate the association of psychosocial working conditions with trajectories of disability. Further studies are required to explore more mechanisms that underlie the association between psychosocial working conditions and disability trajectories.

## Abstract 2

Kuan-Yu Pan and colleagues reveal potential protective effects of psychosocial support at work against cognitive decline and disability.

## Author summary

**Why was this study done?:**

- Work is one of the activities that take up a considerable amount of time in our adult lives, thus potentially making it an important determinant of health, even in later life.
- Inability to independently carry out daily tasks (defined as disability) can affect older people’s quality of life and pose a burden on caregivers and societies.
- A better understanding of the pathway between midlife working conditions and late-life disability may help the development of preventive strategies.

**What did the researchers do and find?:**

- We studied the association of psychosocial working conditions with the rate of disability progression over 12 years in a cohort of 2,937 individuals aged 60 years and older.
- We found that unfavorable psychosocial working conditions, including high-strain, low-strain, and passive jobs, were related to a faster rate of disability progression. The association of high-strain jobs with accelerated disability accumulation was only present among people with low social support at work.
- The decrement in cognitive function and increase in chronic-disease burden, and especially the former, partially explained the relationship between unfavorable working conditions and disability progression in later life.

**What do these findings mean?:**

- Unfavorable psychosocial working conditions during working life are related to the progression of disability in later life. Public health authorities, employers, and employees should all be aware of that.
- Social support at work is especially important in a high-strain work environment given its capacity to attenuate the impact of high-strain jobs on disability accumulation.
- Monitoring cognitive function and medical conditions of people with unfavorable working conditions is endorsed by the role of both dimensions, and especially of cognitive dysfunction, in accelerating disability progression in older age.

## Introduction

Adults aged 60 years or over are one of the faster growing segments of the population worldwide, and they are projected to reach 2 billion by 2050, with a major rise in the number of the oldest old (aged 85 years or over) [1,2]. Advanced age is associated with progressive increase in the occurrence of functional impairment and disability, as a result of both age-related and disease-driven mechanisms. Environmental exposures can affect the accrual rate of disability, and they could thus be modulated to prevent or delay its onset and progression.

Work is one of the main activities that take up a great deal of time in our adult lives, and thus working conditions may play a significant role in influencing people’s health. There is increasing evidence showing that negative psychosocial working conditions can impact both mental and physical health beyond working life (i.e., after retirement) [3,4], which may result in subsequent development of disability later in life. One of the well-established models to assess psychosocial conditions in work environment is the demand–control model [5], which conceptualizes four work scenarios: high strain (high demands/low control), low strain (low demands/high control), passive job (low demands/low control), and active job (high demands/high control). This model was further developed with the inclusion of social support at the workplace [6]. Thus, iso-strain, the combination of high strain and low social support at work, was additionally introduced and suggested to be the most detrimental work scenario, whereas high social support may buffer the negative impact of high strain on health.

Previous studies regarding occupational activities and disability in old age have focused on occupational characteristic [7] and physical demands [8], leaving the association between psychosocial work status and late-life disability mostly unexplored. To our knowledge, only one study showed that high strain and passive jobs were related to a higher number of disabilities in activities of daily living (ADL; including dressing, bathing, toileting, eating, transferring, and continence) and instrumental ADL (IADL; including grocery shopping, meal preparation, laundry, housekeeping, managing medication, handling money, using telephone, and taking public transportation) 28 years after the work experience [9]. However, this study did not capture the change in disability over time. In previous studies, we reported an accelerated rate of cognitive decline [10] and multimorbidity progression (i.e., the co-occurrence of chronic diseases) [11] in people with high strain, low strain, or passive jobs compared with those with active jobs. It has been demonstrated that rapid cognitive decline [12] and chronic-disease accumulation [13] accelerated the accumulation of ADL and IADL disabilities in old age, suggesting that the decrement in cognitive function and increased burden of medical conditions may potentially play a role in mediating the association between working conditions and late-life disability.

In the current prospective study, we aimed to examine whether job demand–control status is associated with the rate of disability accumulation in later life, to verify whether social support at work modifies the relationship between high strain and disability progression, and to investigate whether and to what extent the job–disability association is mediated by baseline cognitive function and medical conditions, as well as cognitive decline and chronic-disease accumulation that occurred during the follow-up period.

## Materials and methods

### Study population and data collection

**Fig 1:**
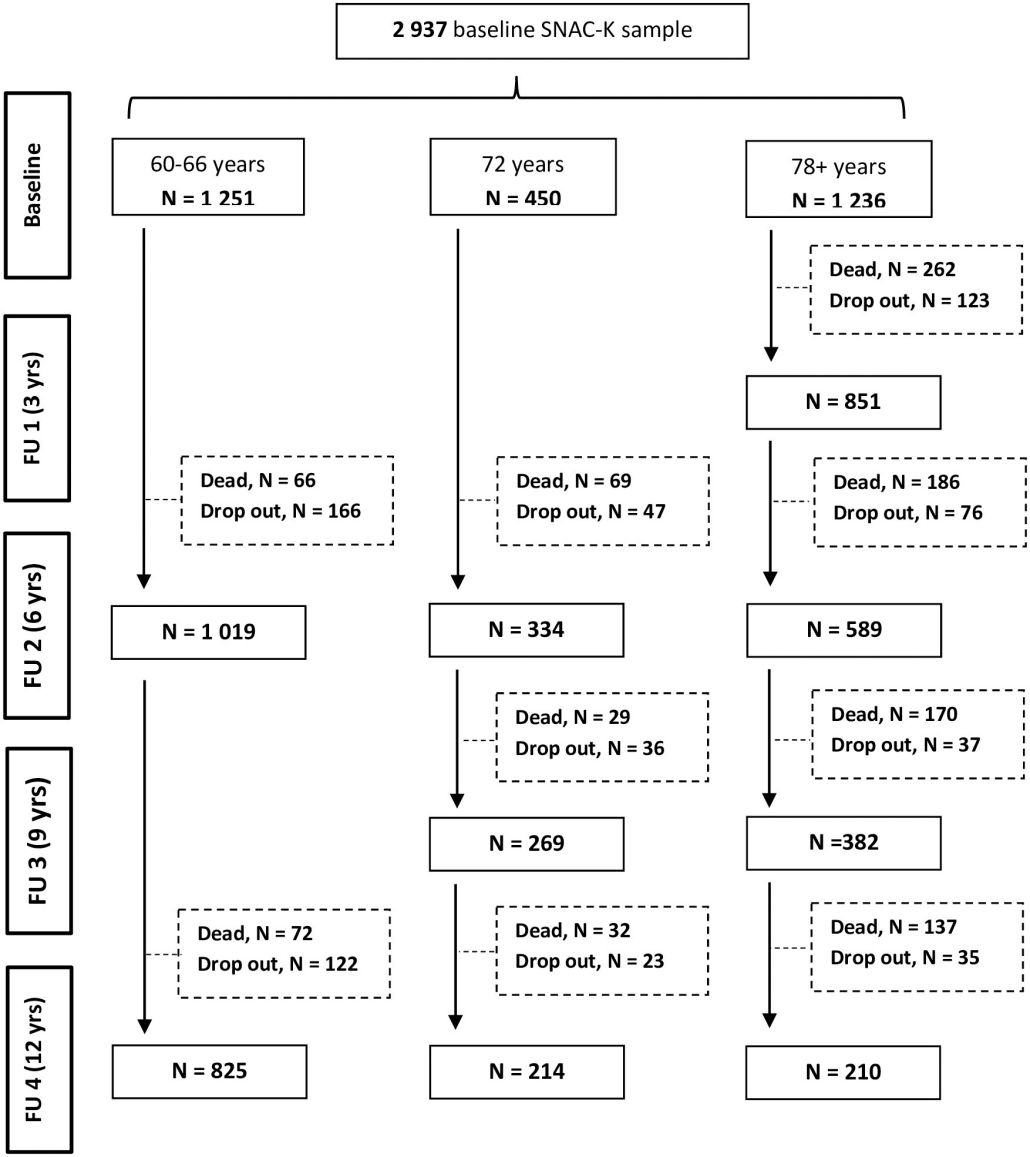
Flowchart of study participation over 12 years. Dropouts are due to either refusal of the participant/relative, loss of contact with the participant, or moving of the participant from the city where the study took place. FU 1, follow-up 1; FU 2, follow-up 2; FU 3, follow-up 3; FU 4, follow-up 4; SNAC-K, Swedish National Study on Aging and Care-Kungsholmen.

Participants of the current study were derived from a population-based observational survey, the Swedish National Study on Aging and Care-Kungsholmen (SNAC-K), in which residents aged 60+ years of the Kungsholmen District in Stockholm were randomly sampled from 11 age cohorts [14]. After the baseline survey (March 2001 to June 2004), the younger-age cohorts (60, 66, and 72 years old) were followed up every 6 years, whereas the older ones (78, 81, 84, 87, 90, 93, 96, and 99+ years old) were followed up every 3 years. Among those who were alive and eligible, 3,363 (73.3%) took part in the baseline examination. People with missing information on occupational history (*n* = 330) or baseline ADL or IADL disabilities (*n* = 96) were excluded, leaving 2,937 participants to be followed up to 12 years. Participants who died or dropped out after the baseline assessment (*n* = 733) were retained in the analyses, contributing exclusively to the baseline association between exposures and outcomes. People who did not have occupational information (10%) and those who died or dropped out after the baseline assessment (25%) were older, less educated, and had more disabilities in both ADL and IADL at baseline. Participants who did not attend follow-up examination were less likely to work in active jobs. Fig 1 depicts the flow of participants over 12 years.

In SNAC-K, data were collected following a structured protocol (http://www.snac-k.se) that consisted of interviews and clinical examinations by nurses and physicians, cognitive assessment by psychologists, and laboratory tests according to standard procedures. For participants with cognitive problems, caregivers or proxies were consulted. Information on medical history was additionally obtained through the linkage to the Swedish National Patient Register; vital status was confirmed by the Swedish Cause of Death Register. Each wave of the SNAC-K study was approved by the Ethical Committee at Karolinska Institutet and the Regional Ethics Review Board in Stockholm, Sweden. Written informed consent was received from participants or next of kin. The results of the present study were reported in accordance with the STROBE guidelines (S1 Checklist).

### Psychosocial working condition

Detailed information on the five longest-held jobs throughout the participants’ professional lives, including employers, job titles, tasks, and time spans, were collected through nurses’ interview at baseline [14]. We first coded each occupation using the three-digit Nordic occupation classification [15]. Then, we merged the occupational codes with a validated psychosocial job–exposure matrix [16] to assess job control, demands, and social support in each occupation. Job control refers to the extent of autonomy to make decisions in conducting work tasks; job demands refer to mental workload and time restriction in completing work tasks; social support at work refers to the general social network at work and help received from coworkers [6,17]. This matrix was constructed based on the Swedish Work Environment Survey and has been used previously in studies on cardiovascular, metabolic, and neurodegenerative diseases [18–20], as well as in our studies on the speed of cognitive decline and chronic-disease accumulation [10,11].

We focused on the longest-held occupation (mean duration 23.5 ± 11.3 years in our study population) because it is deemed as the main contributor to work status throughout the working life. For the operationalization of demand–control status, we dichotomized continuous scores of job control, demands, and support as high/low using the median values from the job–exposure matrix, respectively. We then generated a four-category variable designating psychosocial working conditions, based on demand–control combinations: high strain (high demands/low control), low strain (low demands/high control), passive job (low demands/low control), and active job (high demands/high control). Iso-strain was further defined as the combination of high strain and low social support [6]. The five most prevalent occupations in the SNAC-K cohort by demand–control–support status are listed in Table 1.

**Table 1:** The five most prevalent occupations in the SNAC-K cohort by demand–control–support status.

Demand–control–support status	Occupation
Active job	banker, teacher, engineer, journalist, manager
Passive job	vender, postman, construction worker, graphic printer, cleaner
Low strain	officer, housemaid, home-care assistant, librarian, technician
High strain	cashier, bus driver, pharmacist, dentist assistant, hairdresser
Iso-strain	cashier, bus driver, hairdresser, telephone operator, telephone customer service

Abbreviation: SNAC-K, Swedish National Study on Aging and Care-Kungsholmen

### Disability

The capacity to independently carry out ADL and IADL was reported by participants at baseline and all of the follow-up examinations. Caregivers were also asked to confirm these reports. People living in institutions were assumed to be dependent for grocery shopping, meal preparation, housekeeping, and laundry. ADL and IADL disabilities were treated as separate outcomes first, followed by summing the numbers of ADL and IADL limitations in one scale to enhance the range and sensitivity of disability measurement [21].

### Global cognitive function

Global cognitive function was assessed using a 30-point version of the Mini-Mental State Examination (MMSE) [22] at baseline for all participants, in three occasions for the older cohorts (3-, 6-, and 9-year), and in one occasion for the younger ones (6-year) during the follow-up.

### Chronic disease

Chronic medical conditions were ascertained by clinical examination, medical history, medication use, laboratory data, linkage to inpatient and outpatient care data from the Swedish National Patient Register, and/or through proxy interviews [23]. All diagnoses were coded in accordance with the *International Classification of Diseases*, *10th revision* (ICD-10). Chronic diseases were defined as permanent conditions and when either (1) residual disability remained after the end of the clinical episode or quality of life was worsened compared with the period before the onset of the diseases or (2) long-term (including both regular and intermittent) care, treatment, or rehabilitation was required. A total of 918 ICD-10 codes were identified and classified into 60 chronic-disease categories [13,23]. The total number of chronic diseases was calculated at the same occasions when the MMSE was conducted. We used 6–9-year follow-up data on the mediators (i.e., MMSE score and number of chronic diseases) and 12-year follow-up data on the outcome (i.e., disability) to address the issue of temporality between the mediators and the outcome.

### Covariates

Educational level was recorded as elementary, high school, and university according to the highest degree achieved. Smoking was dichotomized as never/former and current smoking. Alcohol consumption was categorized into none or occasional, light to moderate, and heavy drinking [24]. Engagement in global leisure activities (including mental, social, and physical) was rated as low, moderate, and high, as reported previously [25]. Early-life socioeconomic status (SES) was assessed using occupation of participant’s father, which was categorized into manual, intermediate, and professional [26]. To disentangle psychosocial working condition from other components of the occupation that may also be related to disability, we took into account the occupational characteristic (blue- and white-collar workers) as well as physical demands (light, moderate, and strenuous) in the longest-held job [8].

### Statistical analysis

The study protocol and analysis plan are reported in Supporting information (S1 Study Protocol). Linear mixed-effects models were used to investigate the association between job demand–control status and change in the number of disabilities, using follow-up time (years) as timescale. The fixed effect included demand–control status, follow-up time, and their interaction. The random effect took into consideration individual differences in disability at baseline (random intercept) and in the rate of disability accumulation (random slope). The estimated effect of the interaction term reflects the impact of demand–control status on annual change in the number of disabilities. We converted the original ratings of demands and control into ascending scales, respectively (i.e., higher scores represent greater demands/control), to facilitate interpretation. Job control and demands were analyzed both as continuous and dichotomous variables. Furthermore, we compared the association of each demand–control category with the rate of disability accumulation, using active job as reference [10,18]. Statistical interaction between high strain and low social support in estimating disability progression was tested by introducing a three-way interaction term (i.e., job strain, social support, and time) in the model first, followed by stratified analyses by level of social support at work.

To test and quantify the mediating effects of baseline cognition and medical conditions as well as changes in global cognitive function and chronic-disease burden during the follow-up on the association between demand–control status and rate of disability accumulation, mediation analyses were performed in two separate models: (1) only the baseline MMSE score and number of chronic diseases were treated as mediators; (2) the changes in MMSE score and number of chronic diseases were treated as mediators, and the baseline MMSE score and number of chronic diseases were adjusted for as covariates. In additional analyses, the number of chronic diseases was reduced by one unit at each wave of follow-up if participants had MMSE scores below 24 and were newly diagnosed with incident dementia (i.e., dementia is one of the 60 chronic diseases considered in the total count) to disentangle the mediating effects of the decrease of MMSE score and dementia diagnosis.

In the aforementioned analyses (including linear mixed-effects models and mediation analyses), we first adjusted for age, sex, and education (model 1) and additionally controlled for baseline smoking, alcohol consumption, leisure activity engagement, early-life SES, occupational characteristic, and physical demands in the longest-held job (model 2). Baseline number of chronic diseases and MMSE score were also adjusted for in linear mixed-effects models. In sensitivity analyses, we excluded (1) those who had at least one ADL or IADL disability (*n* = 538), to restrict the study sample to those without any disability at baseline; (2) those who were still working at baseline (*n* = 582), to prevent potential reversed causation; and (3) prevalent dementia cases and incident dementia during the first 3 years of follow-up (*n* = 153), and we assessed work status by the latest job to reduce potential information bias because of poor cognition. Finally, to allow for comparability of results across models 1 and 2, we conducted multiple imputation by chained equation for missing data in covariates, obtaining five imputed datasets [27]. The estimates of these datasets were pooled according to Rubin’s rule for valid statistical inferences. All analyses were computed using Stata SE 15.0 (StataCorp, College Station, TX, United States).

## Results

Baseline characteristics of the study population by demand–control categories are presented in Table 2.

**Table 2:** Baseline characteristics of study population by demand–control status of the longest-held job.

Characteristics	Active job	Low strain	High strain	Passive job
	*n* = 1,807	*n* = 495	*n* = 328	*n* = 307
Age (years)	71 ± 10	76 ± 10	74 ± 11	78 ± 11
Female sex	1,035 (57.3)	375 (75.8)	224 (68.3)	212 (69.1)
Education				
Elementary	128 (7.1)	106 (21.4)	74 (22.6)	160 (52.1)
High school	808 (44.7)	321 (64.9)	190 (57.9)	129 (42.0)
University	871 (48.2)	68 (13.7)	64 (19.5)	18 (5.9)
Occupational characteristic				
Blue collar	88 (4.9)	202 (40.8)	138 (42.1)	244 (79.5)
White collar	1,719 (95.1)	293 (59.2)	190 (57.9)	63 (20.5)
Occupational physical demands				
Light	1,532 (87.2)	302 (63.4)	209 (65.9)	136 (45.5)
Moderate	172 (9.8)	106 (22.3)	85 (26.8)	99 (33.1)
Strenuous	53 (3.0)	68 (14.3)	23 (7.3)	64 (21.4)
Number of chronic diseases*	3 (2, 5)	4 (3, 6)	4 (2, 5)	4 (3, 6)
MMSE score	28.9 ± 2.7	27.8 ± 3.2	28.5 ± 4.9	26.8 ± 4.7
ADL disability^§^	59 (3.3)	48 (9.7)	16 (4.9)	33 (10.8)
IADL disability^μ^	242 (13.4)	119 (24.0)	73 (22.3)	83 (27.0)

Data are presented as mean ± standard deviations or number (proportion %). Number of people with missing data for occupational physical demands: 88.

*Data are presented as medians and interquartile ranges.

^§^At least one impairment in ADL.

^μ^At least one impairment in IADL.

Abbreviations: ADL, activities of daily living; IADL, instrumental ADL; MMSE, Mini-Mental State Examination

Low levels of job demands and control were associated with a higher annual increase in the number of total (ADL + IADL) disabilities, regardless of whether job demands and control were treated as continuous or dichotomous variables (Table 3). Similar results were found in the analyses in which ADL and IADL limitations were treated as separate outcomes (S1 Table).

**Table 3:** Association of job demands and control with annual change in the number of total (ADL + IADL) disabilities over 12 years.

Job demands/control × time	Model 1^a^			Model 2^b^		
	β	95% CI	*p*	β	95% CI	*p*
Job control (continuous)	−0.03	−0.05 to −0.02	<0.001	−0.04	−0.05 to −0.02	<0.001
Job demands (continuous)	−0.07	−0.09 to −0.04	<0.001	−0.08	−0.10 to −0.05	<0.001
Job control (dichotomous)						
High	Ref.			Ref.		
Low	0.06	0.02–0.09	0.001	0.07	0.03–0.12	0.001
Job demands (dichotomous)						
High	Ref.			Ref.		
Low	0.04	0.01–0.07	<0.05	0.10	0.06–0.14	<0.001

^a^Adjusted for age, sex, and education.

^b^Adjusted for age, sex, education, alcohol consumption, smoking, leisure activity engagement, early-life socioeconomic condition, occupational characteristic and physical demands, and baseline number of chronic diseases and MMSE score.

Abbreviations: ADL, activities of daily living; IADL, instrumental ADL; MMSE, Mini-Mental State Examination; Ref, reference group

**Fig 2:**
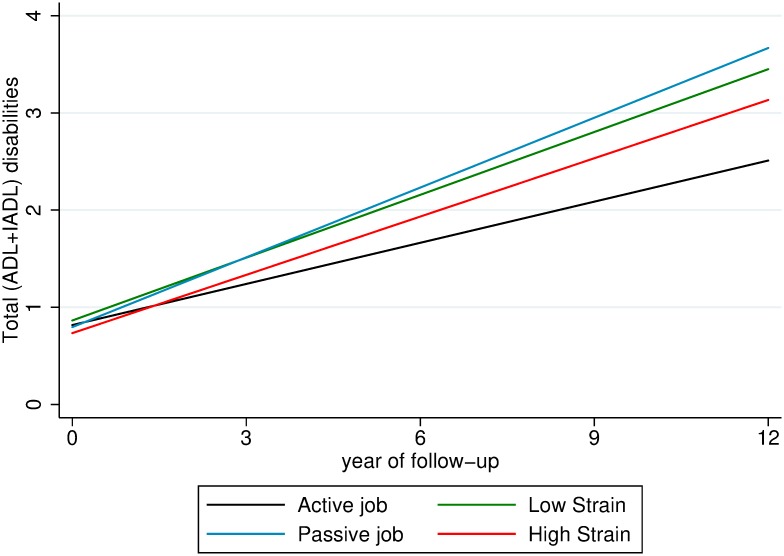
Trajectories of total (ADL + IADL) disabilities over 12 years by demand–control status. Trajectories derived from linear mixed-effects model adjusted for age, sex, education, alcohol consumption, smoking, leisure activity engagement, early-life socioeconomic condition, occupational characteristic and physical demands, and baseline number of chronic diseases and MMSE score. Reference group: active job. ADL, activities of daily living; IADL, instrumental ADL; MMSE, Mini-Mental State Examination.

Fig 2 shows the trajectories of total (ADL + IADL) limitations over time associated with different demand–control categories. Relative to active jobs, high strain (β = 0.07, 95% CI 0.02–0.13), low strain (β = 0.10, 95% CI 0.06–0.15), and passive jobs (β = 0.11, 95% CI 0.05–0.18) were all related to an accelerated speed of total (ADL + IADL) disability accumulation during the follow-up. Similarly, high strain (β = 0.03, 95% CI 0.01–0.05; β = 0.04, 95% CI 0.01–0.08), low strain (β = 0.02, 95% CI 0.003–0.04; β = 0.08, 95% CI 0.05–0.11), and passive jobs (β = 0.03, 95% CI 0.01–0.06; β = 0.09, 95% CI 0.05–0.13) were associated with faster progression of ADL disability and IADL disability, respectively (S1 Fig). No association between demand–control status and baseline disability was observed. Considering the same trend across results with respect to ADL, IADL, and total (ADL + IADL) disabilities, the following analyses focused on total (ADL + IADL) disabilities.

We detected a multiplicative interaction (i.e., a statistically significant interaction in a multiplicative scale) between high strain and low social support at work in relation to change in the number of total (ADL + IADL) disabilities (*p <* 0.05). In the subsequent stratified analyses by social support, we observed that the association between high strain and total disability accumulation was only present among workers with low social support, but not among those with high social support (Table 4). Sensitivity analyses produced similar results.

**Table 4:** Association of demand–control status with annual change in the number of total (ADL + IADL) disabilities over 12 years by level of social support at work.

Strata		Model 1^a^			Model 2^b^		
Demand–control × time	*n*	β	95% CI	*p*	β	95% CI	*p*
**High social support**							
Active job	697	Ref.			Ref.		
Low strain	410	0.08	0.01–0.14	<0.05	0.09	0.02–0.15	<0.01
High strain	157	−0.001	−0.10 to 0.09	0.98	0.004	−0.09 to 0.10	0.93
Passive job	252	0.09	0.003–0.17	<0.05	0.09	0.01–0.17	<0.05
**Low social support**							
Active job	1,110	Ref.			Ref.		
Low strain	85	0.05	−0.04 to 0.15	0.28	0.09	−0.01 to 0.18	0.06
High strain	171	0.12	0.06–0.19	<0.001	0.13	0.07–0.19	<0.001
Passive job	55	0.09	−0.02 to 0.21	0.12	0.09	−0.03 to 0.20	0.15

^a^Adjusted for age, sex, and education.

^b^Adjusted for age, sex, education, alcohol consumption, smoking, leisure activity engagement, early-life socioeconomic condition, occupational characteristic and physical demands, and baseline number of chronic diseases and MMSE score.

Abbreviations: ADL, activities of daily living; IADL, instrumental ADL; MMSE, Mini-Mental State Examination; Ref, reference group

**Fig 3:**
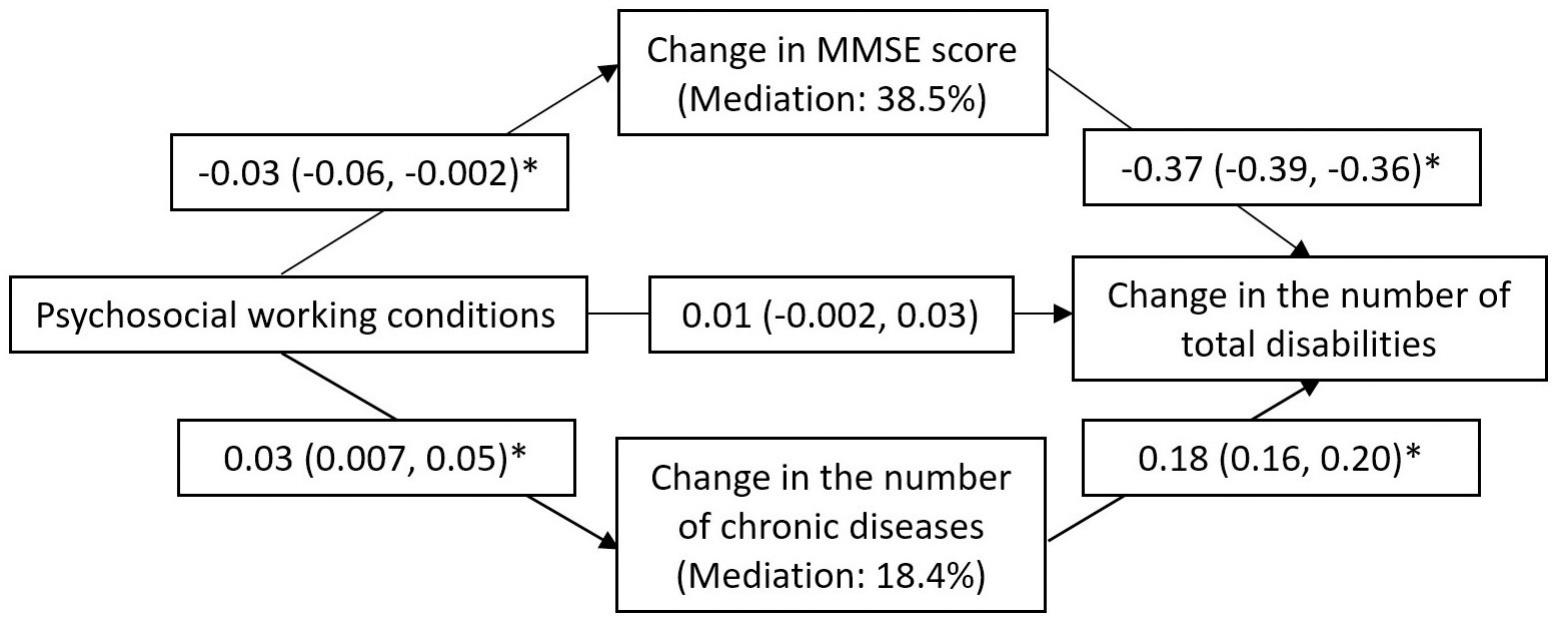
Mediation of changes in cognitive function and chronic-disease burden in the work–disability association. Mediation model adjusted for age, sex, education, alcohol consumption, smoking, leisure activity engagement, early-life socioeconomic condition, occupational characteristic and physical demands, and baseline number of chronic diseases and MMSE score. *p < 0.05. MMSE, Mini-Mental State Examination.

In the mediation analyses, demand–control category was treated as an ordinal variable whose detrimental effect increased from the lowest to the highest according to the magnitude of associations shown in Fig 2: active job, high strain, low strain, and passive job. Such dose-response pattern was also seen with respect to changes in MMSE score and number of chronic diseases over time [10,11]. The mediation analyses revealed that adverse psychosocial working conditions were associated with a greater decrease in MMSE score and a greater increase in the number of chronic conditions over time, both of which were in turn related to a faster speed of total (ADL + IADL) disability accumulation. After controlling for a range of potential confounders, 38.5% of the association between demand–control status and total (ADL + IADL) disability trajectories was mediated by the decline in cognitive function, whereas 18.4% was mediated by the accumulation of medical conditions that occurred during the follow-up period (Fig 3). Baseline cognitive function and medical conditions did not mediate the association between demand–control status and disability progression. Additional analyses addressing the issue of the overlapping effects of excessive decrease in MMSE score and dementia diagnosis showed the same results.

## Discussion

In this population-based cohort study, low levels of job control and demands were associated with a higher rate of disability progression among individuals aged 60+ years over 12 years of follow-up. By applying the categorization of the demand–control model, we found that in comparison with active jobs, high strain, low strain, and passive jobs were all related to an accelerated speed of disability progression. When stratified by social support, the association between high strain and disability accumulation was only shown in people with low social support at work (i.e., iso-strain), but not in those with high social support. These results remained even after taking into consideration several possible confounders including occupational characteristic and work-related physical demands. Moreover, the association between work-related psychosocial status and disability trajectories was partially mediated by both cognitive decline (38.5%) and accumulation of chronic diseases (18.4%).

Active jobs are usually a professional type of work, and people who undertake these jobs may have many advantageous aspects of development and lifestyle throughout the life course [5]. Indeed, participants with active jobs in the current study came from families of better SES, had higher educational attainment, tended to be more engaged in leisure activities, and possessed a richer social network in the societal context. Despite the adjustment for these factors, the possibility of uncontrolled residual confounding could not be ruled out. Nevertheless, there are potential physiological mechanisms that underlie the observed association between psychosocial work status and disability trajectories. These mechanisms likely differ across demand–control categories and could have been partly clarified by the mediation analyses conducted here. High strain, as well as iso-strain, represents stressful work scenarios. With repeated exposure to stress over prolonged duration, dysregulated hypothalamus–pituitary–adrenal cortex axis induces hypersecretion of glucocorticoid hormone [28], which could further affect neurons in the brain and cognition [29,30]. In addition, chronic stress has been linked to elevated allostatic load, which compromises homeostatic capacity [31]. The rapid accumulation of multimorbidity could be the consequence of a high degree of cellular and molecular damage, which may gradually chip away at physiological reserve [32,33], subsequently contributing to the depleted physical and cognitive functioning [34]. By contrast, low strain, a relaxing work status, may not provide sufficient mental exercise, which, according to the cognitive reserve theory, could lead to cognitive decrement, several neurodegenerative conditions, and thereafter, functional limitations [35,36]. Finally, passive jobs, the combination of low control and low demands, have been argued as both mentally unchallenging and stressful because of lack of development and self-efficacy [37], which might have explained the greatest effect magnitude on disability progression from this construct observed here.

The findings of associations of high strain and passive jobs with a faster speed of late-life disability progression are in line with the study that demonstrated more ADL and IADL limitations in older adults with these occupations during working lives [9]. Similarly, high strain and passive jobs as well as one of their components, low job control, have all been linked to work disability [38–41] and poor functioning in old age [3,4,42]. However, studies regarding job demands reported discrepant results, which may be due to varying occupation characteristics. One Swedish study showed that high demands were associated with a higher likelihood of receiving a disability pension among the general population with all sorts of jobs [39], whereas a Finnish study found no association between job demands and retirement due to disability among employees working in the public sector [41]. In the present study, we observed that low job demands were related to a more rapid progression of disabilities in old age.

Furthermore, social support at work served as an effect modifier in the association between high strain and disability accumulation in our study. We detected an interactive effect of high strain and low social support on disability progression, whereas high strain was not associated with disability in people with high social support. Similarly, in our previous study, the association between high strain and accelerated cognitive decline was only shown in people with low social support, but not in those with high social support [10]. Iso-strain, the combination of high strain and low social support, has been proposed as the most detrimental work scenario [6] and has been related to a higher risk of sick leave [43], anxiety, and depression [44]. On the other hand, high strain was no longer detrimental when social support at work was sufficient. Therefore, our findings highlight the importance of work-related social support, especially in a stressful work environment.

The chain of risk model from the life-course perspective asserts a sequence of linked factors that lead to health outcomes [45]. Thus, the mediation analyses in the current study may have helped in the understanding of potential mechanisms linking working conditions and disability progression in old age. Of the total association, we found that the mediating effect of cognitive decline was more than two times greater than that of chronic-disease accumulation (38.5% versus 18.4%). This may suggest that cognitive dysfunction plays a more relevant role than physical depletion in explaining the impact of psychosocial working conditions on the progression of functional limitations. It is nevertheless noteworthy that these findings were based on an assumption that the observed associations of psychosocial aspects of work environment with later-life disability were causal. In addition, cognitive decline and chronic conditions may interact and further affect disability later on [46]. The mediating effect of such interaction has not been captured by our analyses. However, the correlation between changes in cognitive function and number of diseases in our study population was relatively small (*r* = −0.32).

The strengths of this study include the use of population-based data and prospective design in terms of the repeated measurements of outcome (i.e., number of ADL and IADL limitations) and mediating variables (i.e., MMSE score and number of chronic diseases) at baseline as well as at each wave of the follow-up. The occupational information throughout working life enabled assessment of working conditions from different chronological perspectives, including the longest-held jobs and the latest jobs. The inclusion of both ADL and IADL could help capture a wider range of limitations in tasks of daily living. Other occupational components (i.e., characteristic and physical demands) and individual predispositions (i.e., education and early-life SES) that might preselect participants into occupations were also taken into consideration.

The following limitations should be considered. First, retrospective recall of occupational experience throughout working life could lead to misclassification; nevertheless, the sensitivity analyses may have addressed this issue. Second, reliance on a psychosocial job–exposure matrix does not consider variabilities in individuals’ perception on working conditions or job characteristics within occupations. However, self-reporting bias should have been reduced by this approach. Third, the lack of data from participants who dropped out during the follow-up may affect the results. However, had we been able to follow them, the magnitude of the associations reported here would presumably have been greater. Fourth, we intended to address the issue of temporality between the mediators (i.e., MMSE score and chronic diseases) and the outcome (i.e., disability) by using 6–9-year follow-up data on the mediators and 12-year follow-up data on the outcome. However, the assessments of the mediators and outcome might have taken place in the same occasions; thus, the reversed causation between them may not be completely ruled out. Finally, the SNAC-K study population is of relatively higher socioeconomic background, which may have diluted the observed association between working conditions and disability.

In conclusion, psychosocial working conditions during working life, characterized by high strain, low strain, and passive jobs, are associated with a faster rate of disability progression in later life, and the change in cognitive function seems to play a more relevant role in this association than the change in chronic-disease burden. Notably, high social support at work may buffer the detrimental impact of high strain on disability progression. Our findings highlight the importance of the psychosocial component of work, beyond occupational characteristic and physical demands, in determining the reserve of functional ability in old age.

## Supporting information

S1 Checklist STROBE checklist. STROBE, strengthening the reporting of observational studies in epidemiology.
(DOCX)

S1 Study Protocol Study protocol. (DOCX)

S1 Table Association of job demands and control with annual change in ADL disability and IADL disability over 12 years. Adjusted for age, sex, education, alcohol consumption, smoking, leisure activity engagement, early-life socioeconomic condition, occupational characteristic and physical demands, and baseline number of chronic diseases and MMSE score. ADL, activities of daily living; IADL, instrumental ADL; MMSE, Mini-Mental State Examination; Ref, reference group.
(DOCX)

S1 Fig Trajectories of ADL disability and IADL disability over 12 years by demand–control status. Trajectories derived from linear mixed-effects model adjusted for age, sex, education, alcohol consumption, smoking, leisure activity engagement, early-life socioeconomic condition, occupational characteristic and physical demands, and baseline number of chronic diseases and MMSE score. Reference group: active job. ADL, activities of daily living; IADL, instrumental ADL; MMSE, Mini-Mental State Examination.
(DOCX)

## Abbreviations

ADL: activities of daily living
IADL: instrumental ADL
ICD-10: *International Classification of Diseases*, *10th revision*
MMSE: Mini-Mental State Examination
SES: socioeconomic status
SNAC-K: Swedish National Study on Aging and Care-Kungsholmen
