# Supplementary material for: Psychosocial working conditions, trajectories of disability, and the mediating role of cognitive decline and chronic diseases: A population-based cohort study
Source: PLoS Med. 2019 Sep 16;16(9):e1002899. doi: 10.1371/journal.pmed.1002899 (PMC6746356; doi:10.1371/journal.pmed.1002899)
Supplement: S1 Study Protocol — (DOCX) [file pmed.1002899.s002.docx]

**S1 Study Protocol**

The present study is part of the four-year PhD project of the first author, which has been approved by the student’s supervisors and by the Committee for Doctoral Education of the Department of Neurobiology, Caring Sciences and Society of Karolinska Institutet, Sweden. The title of the PhD project is “*Impact of psychosocial working conditions on health in older age.*” The study in question was conceived in October 2018, following the previous publication by the first author, and building on the ongoing work in our research group at the Aging Research Center (see below for detail).

1. Pan KY, Xu W, Mangialasche F, Dekhtyar S, Fratiglioni L, Wang HX. Working life psychosocial conditions in relation to late-life cognitive decline: a population-based cohort study. Journal of Alzheimer's disease: JAD. 2019; 67:315-325
2. Dekhtyar S, Vetrano DL, Marengoni A, Wang HX, Pan KY. Fratiglioni L, et al. Association between speed of multimorbidity accumulation in old age and life experiences: a cohort study. American journal of epidemiology. 2019; kwz101, <https://doi.org/10.1093/aje/kwz101>. Published: 24 April 2019.
3. Calderon-Larranaga A, Santoni G, Wang HX, Welmer AK, Rizzuto D, Vetrano DL, et al. Rapidly developing multimorbidity and disability in older adults: does social background matter? Journal of internal medicine. 2018;283(5):489-99

Below we report the analysis plan of the current study.

**Analysis Plan:**

1. Hypotheses:
2. Job demand-control status is associated with the rate of disability accumulation in later life.
3. Social support at work can serve as an effect modifier in the relationship between high strain and disability accumulation.
4. Cognitive decline and chronic disease accumulation that occurred during the follow-up period can mediate the link of demand-control status to disability trajectory.
5. Planned analytical approach:
6. Performing linear mixed-effects models to investigate the association of job demands, job control, and demand-control status with the rate of disability accumulation, using follow-up time (years) as time scale.
7. Testing statistical interaction between high strain and low social support in estimating disability trajectory by introducing a three-way interaction term (i.e., job strain, social support, and time) in the model. In case of a statistically significant interaction detected, stratified analyses by levels of social support at work will be further carried out.
8. Performing mediation analyses where the MMSE score and number of chronic diseases are treated as time-varying variables.
9. Analysis that was actually performed:
10. Linear mixed-effects models to investigate the association of job demands, job control, and demand-control status with the rate of disability accumulation.
11. Models testing the interaction between high strain and low social support on disability trajectory.
12. Stratified analyses by levels of social support at work.
13. Mediation analyses in two separate models: 1) only the baseline MMSE score and number of chronic diseases were treated as mediators; 2) the changes in MMSE score and number of chronic diseases were treated as mediators and the baseline MMSE score and number of chronic diseases were adjusted for as covariates.
14. Deviations between planned and performed analyses:

The only deviation concerns the mediation analyses. In order to examine the mediating role of cognitive function and chronic disease burden in the association between working conditions and disability progression more explicitly, we disentangle the mediating effect of baseline MMSE score and number of chronic diseases from the changes in MMSE score and number of chronic diseases.
