# Supplementary material for: Psychosocial working conditions, trajectories of disability, and the mediating role of cognitive decline and chronic diseases: A population-based cohort study
Source: PLoS Med. 2019 Sep 16;16(9):e1002899. doi: 10.1371/journal.pmed.1002899 (PMC6746356; doi:10.1371/journal.pmed.1002899)
Supplement: S1 Table — Adjusted for age, sex, education, alcohol consumption, smoking, leisure activity engagement, early-life socioeconomic condition, occupational characteristic and physical demands, and baseline number of chronic diseases and MMSE score. ADL, activities of daily living; IADL, instrumental ADL; MMSE, Mini-Mental State Examination; Ref, reference group. (DOCX) [file pmed.1002899.s003.docx]

**S1 Table. Association of job demands and control with annual change in ADL disability and IADL disability over 12 years**

| Job demands/control х time |  | Model 1^a^ |  |  |  | Model 2^b^ |  |
| --- | --- | --- | --- | --- | --- | --- | --- |
|  | β | 95% CI | *p* |  | β | 95% CI | *p* |
| **ADL disability** |  |  |  |  |  |  |  |
| Job control (continuous) | -0.01 | -0.02 to -0.004 | 0.001 |  | -0.01 | -0.01 to -0.002 | <0.01 |
| Job demands (continuous) | -0.02 | -0.03 to -0.005 | <0.01 |  | -0.02 | -0.03 to -0.01 | <0.001 |
| Job control |  |  |  |  |  |  |  |
| high | Ref. |  |  |  | Ref. |  |  |
| low | 0.02 | 0.006 to 0.03 | <0.01 |  | 0.03 | 0.01 to 0.04 | <0.01 |
| Job demands |  |  |  |  |  |  |  |
| high | Ref. |  |  |  | Ref. |  |  |
| low | 0.01 | -0.005 to 0.02 | 0.25 |  | 0.02 | 0.01 to 0.04 | <0.01 |
| **IADL disability** |  |  |  |  |  |  |  |
| Job control (continuous) | -0.03 | -0.04 to -0.02 | <0.001 |  | -0.02 | -0.03 to -0.01 | <0.001 |
| Job demands (continuous) | -0.06 | -0.07 to -0.04 | <0.001 |  | -0.06 | -0.08 to -0.04 | <0.001 |
| Job control |  |  |  |  |  |  |  |
| high | Ref. |  |  |  | Ref. |  |  |
| low | 0.04 | 0.01 to 0.06 | <0.01 |  | 0.04 | 0.01 to 0.07 | <0.05 |
| Job demands |  |  |  |  |  |  |  |
| high | Ref. |  |  |  | Ref. |  |  |
| low | 0.03 | 0.01 to 0.05 | <0.01 |  | 0.07 | 0.05 to 0.10 | <0.001 |

^a^ Adjusted for age, sex, and education.

^b^ Adjusted for age, sex, education, alcohol consumption, smoking, leisure activity engagement, early-life socioeconomic condition, occupational characteristic and physical demands, and baseline number of chronic diseases and MMSE score.
