# Supplementary material for: Psychosocial working conditions, trajectories of disability, and the mediating role of cognitive decline and chronic diseases: A population-based cohort study
Source: PLoS Med. 2019 Sep 16;16(9):e1002899. doi: 10.1371/journal.pmed.1002899 (PMC6746356; doi:10.1371/journal.pmed.1002899)
Supplement: S1 Fig — Trajectories derived from linear mixed-effects model adjusted for age, sex, education, alcohol consumption, smoking, leisure activity engagement, early-life socioeconomic condition, occupational characteristic and physical demands, and baseline number of chronic diseases and MMSE score. Reference group: active job. ADL, activities of daily living; IADL, instrumental ADL; MMSE, Mini-Mental State Examination. (DOCX) [file pmed.1002899.s004.docx]

| **ADL disability**  **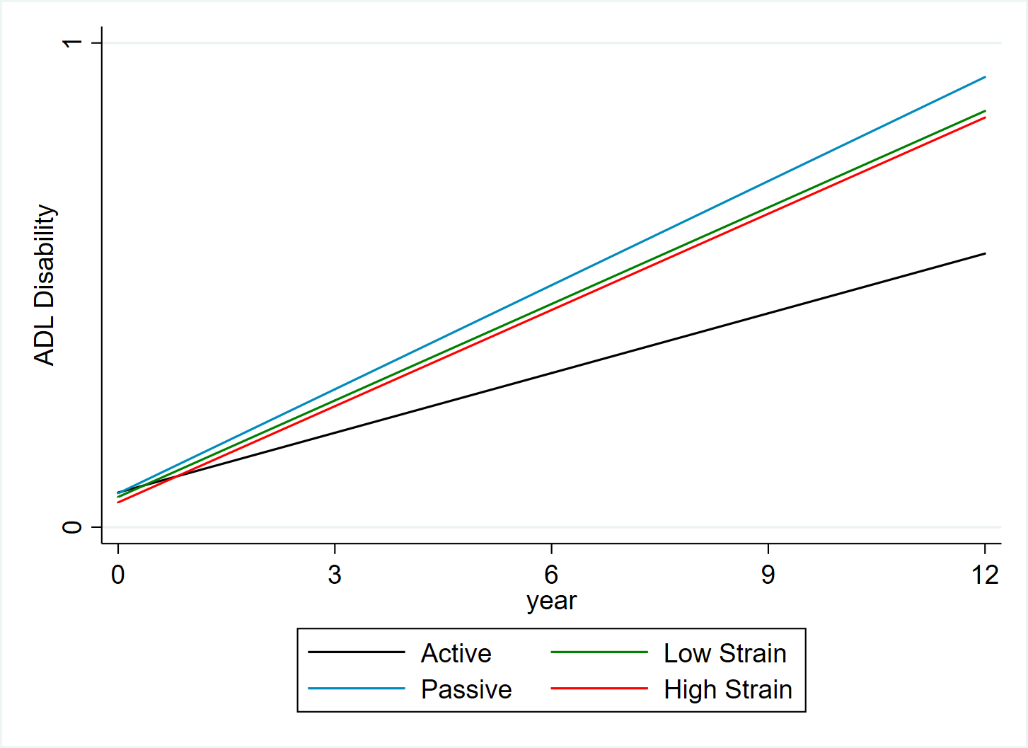** |
| --- |
| **IADL disability**  **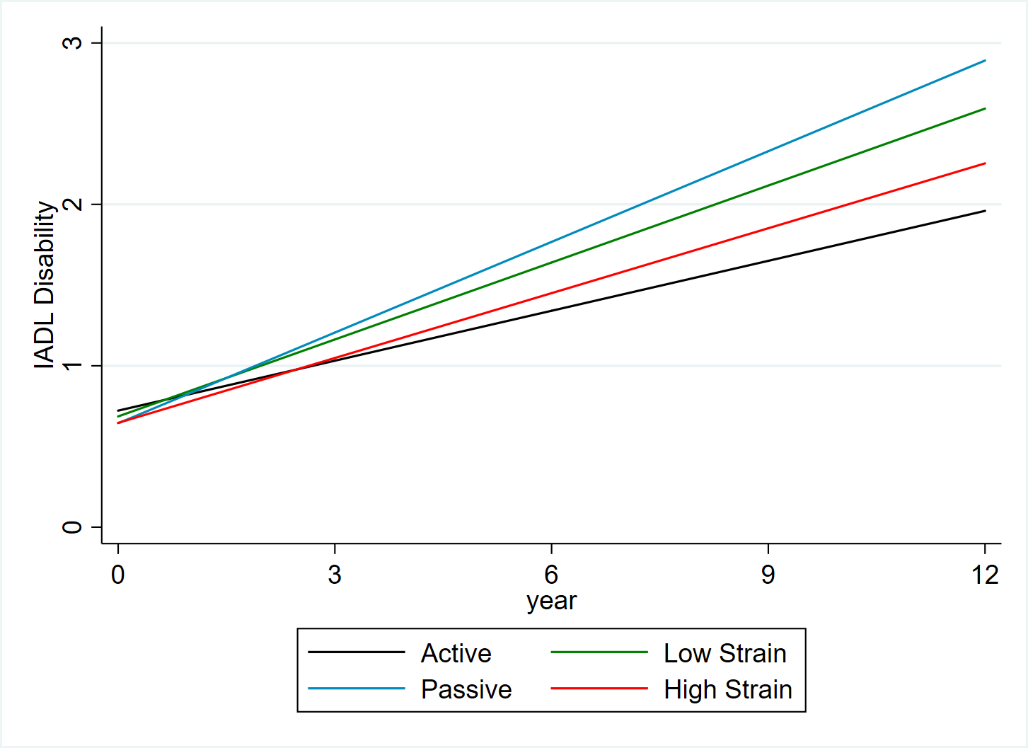** |

**S1 Fig. Trajectories of ADL disability and IADL disability over 12 years by demand-control status.** Trajectories derived from linear mixed-effects model adjusted for age, sex, education, alcohol consumption, smoking, leisure activity engagement, early-life socioeconomic condition, occupational characteristic and physical demands, and baseline number of chronic diseases and MMSE score. Reference group: active job. ADL, activities of daily living; IADL, instrumental activities of daily living.
